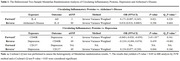# Genetics of circulating inflammatory proteins identifies a potential pathway linking Alzheimer's disease and depression

**DOI:** 10.1002/alz70856_098981

**Published:** 2025-12-24

**Authors:** Hexiao Ding, Gerald Wai‐Yeung Cheng, Hongzhao Chen, Jung Sun Yoo

**Affiliations:** ^1^ Hong Kong Polytechnic University, Kowloon, Kowloon, Hong Kong

## Abstract

**Background:**

Alzheimer's disease (AD) and depression are highly comorbid and share related inflammatory etiologies. Many studies have reported changes in pro‐inflammatory cytokines in cases of depression and AD, however, these findings are often based on small cohorts or animal models. Mendelian randomization (MR) uses single nucleotide polymorphisms (SNPs) as an instrumental variable to examine causal relationships. We utilized large‐scale genome‐wide association studies (GWAS) data among Europeans to determine the direction and magnitude of the impact of inflammatory processes on susceptibility to depression and AD.

**Method:**

Genetic variants from GWAS for 91 circulating inflammatory plasma proteins (*N* = 14,824), depression (*N* = 500,199), and AD (*N* = 487,511) were used for bidirectional MR using inverse variance weighted (IVW) method. SNPs that were strongly associated with the exposure (*p* <5×10^−8^) were selected and the clump function in PLINK 1.9 was used to identify independent SNPs linked to each exposure (clump r^2^ < 0.001, clump kb < 5000). The SNPs with a strong association to the outcome (*p* < 5×10^−8^) and palindromic SNPs were removed. Also, SNPs with F‐statistics <10 were excluded as weak instrumental variables. Cochran's Q test was conducted to ensure the robustness of the results. The results that yielded a *p*‐value < 0.05 in MR analysis by IVW method and a Cochran's Q test *p*‐value > 0.05 were considered significant.

**Result:**

Seventy‐four proteins were eligible for MR analysis after selection. Forward MR analysis found a protective effect of CD40R on depression (IVW OR [95% CI] =‐0.049 [‐0.038, ‐0.059]) and a risk effect of IL‐8 on AD (IVW OR [95% CI] =‐0.171 [0.097, 0.245]). Reverse MR showed that increased genetic risk of depression is associated with increased CD137 (IVW OR [95% CI] = 0.154 [0.077, 0.230]).

**Conclusion:**

The MR analysis revealed IL‐8 increases the risk of AD. The soluble CD40 receptor protects against the progression of depression. Previous studies have shown IL‐8 induces abnormal expression of CD40 by microglia, which may contribute to the comorbidity of depression and AD. Our results also indicate that depression upregulates CD137 expression, a biomarker recognized for AD progression. Future research can explore the potential of CD137 as a biomarker for the co‐diagnosis of depression and AD.